# Long-Term Improvement of Different Types of Acne Vulgaris Using a Mild Photodynamic Therapy Protocol with BF-200 ALA Gel: A Series of Cases

**DOI:** 10.3390/jcm13092658

**Published:** 2024-05-01

**Authors:** Carlos Serra-Guillén, Beatriz Llombart, Onofre Sanmartín

**Affiliations:** 1 Instituto Valenciano de Dermatología y Estética (IVADE), 46003 Valencia, Spain; 2 Clinica Dermatologica Dr. Onofre Sanmartín, 46930 Valencia, Spain; beatriz.llombart@uv.es (B.L.); osanmartinj@gmail.com (O.S.)

**Keywords:** acne vulgaris, photodynamic therapy (PDT), red light

## Abstract

**Background**: Photodynamic therapy (PDT) can be a promising alternative for patients with acne vulgaris. Our study aimed to evaluate the efficacy and safety of red light photodynamic therapy with BF-200 ALA gel in the treatment of different types of acne vulgaris. **Methods**: We performed a retrospective, observational study of a series of 22 cases. All patients were treated according to a mild PDT protocol. After a careful wash of the affected skin areas, BF-200 ALA gel was applied to the skin in a thin layer and incubated for 30 min, followed by illumination using narrow-spectrum red light (635 nm) at a dose of 4 J/cm^2^. Most patients received one (36.4%), two (27.3%), or three (22.7%) PDT sessions. About a third of the patients received concomitant acne treatment with topical retinoids. **Results**: Patients of 25.1 ± 8.9 years suffering from papulopustular (45.5%), nodular (27.3%), and comedonal acne (27.3%) in the face were included. Irrespective of acne type or severity, 95.5% of patients had good or excellent responses to the treatment with PDT (≥60% lesion clearance). We found no association between concomitant acne medication and the favorable results achieved by PDT. Most patients reported no adverse events (72.7%), except for six patients who experienced erythema. The good efficacy results were maintained over a follow-up period of 12.5 ± 10.8 months. **Conclusions**: In this study, we show that PDT with BF-200 ALA gel and low light dose is an effective and long-lasting option for the treatment of different acne types.

## 1. Introduction

Acne vulgaris (acne) is a chronic inflammatory dermatosis affecting between 50% and 95% of adolescents in Westernized societies and occasionally persists into adulthood [1,2,3]. Of these, 20% to 35% have moderate to severe manifestations [3]. Severe acne involving consequent scarring can have psychological implications such as anxiety and depression [4,5,6].

Acne is characterized by non-inflammatory lesions (open and closed comedones), inflammatory lesions (papules, nodules, pustules, cysts), and residual lesions (atrophic and hypertrophic or keloid scars, erythematous macules), mainly affecting the face, neck, shoulders, upper back and chest [4,7,8,9,10]. The severity of acne is determined by the predominant type of lesion and its extent, which depends on the number of affected areas and the degree of involvement.

To date, several treatment options target different severities and morphologies of acne, either topically (e.g., topical antibiotics, benzoyl peroxide, retinoids) or systemically (e.g., oral antibiotics, hormones, and isotretinoin) [1,3,4,11,12]. However, some of the standard treatments are associated with discouraging side effects. For example, isotretinoin is associated with teratogenicity, making it unsuitable for patients with the desire to have children while on this treatment [13]. Topical agents and systemic antibiotics require daily application, often leading to poor treatment compliance. Alternatives to systemic antibiotics for moderate to severe acne are becoming more important as the numbers of antibiotic-resistant bacteria are rising [14]. In addition, we often find in routine clinical practice that many patients with a clear indication for isotretinoin treatment do not wish to receive this treatment because of their fear of side effects and hematological toxicity. Taking all this into account, alternatives to isotretinoin and systemic antibiotics for moderate to severe acne are necessary in daily clinical practice.

Photodynamic therapy (PDT) is a promising alternative for patients who did not respond to standard treatments or for whom standard treatments are not suitable. PDT addresses several mechanisms that may contribute to the pathogenesis of acne: On the structural level, PDT affects the pilosebaceous unit through the destruction of sebocytes and sebaceous gland lobules and through the inhibition of the sebaceous gland’s function. It also produces an increase in epidermal turnover, thus reducing follicular obstruction. On the cellular level, PDT leads to a downregulation of receptors that trigger inflammation. As PDT also has antimicrobial effects, a reduction of *Cutibacterium acnes* (*C. acnes*) in acne patients treated with PDT is considered possible [15].

In the past years, PDT has been increasingly studied for different types of acne and has already been shown to improve certain types of acne [15,16]. The aim of our study was to evaluate the efficacy of photodynamic therapy in the treatment of different types of acne. Therefore, we performed a retrospective, observational, descriptive study of a series of cases with different acne types using a mild PDT protocol with BF-200 ALA.

## 2. Materials and Methods

### 2.1. Study Design

This was a retrospective, observational study of a series of cases. Patients with acne treated with PDT in two centers were included: Consulta de Dermatología Dr. Onofre Sanmartín and Instituto Valenciano de Dermatología y Estética (IVADE) group. Data was collected between 2017 and 2019. The study was performed in accordance with the Declaration of Helsinki (October 2013, Fortaleza, Brazil) and has been approved by the Corporate Clinical Research Ethics Committee of the Ministry of Health of the Generalitat Valenciana (file number CEICC 2019-27) on 21 April 2019.

### 2.2. Study Population and Parameters

Patients diagnosed with acne vulgaris were treated with PDT at two dermatological clinics (IVADE and Clínica Onofre Sanmartin). Patients who followed the treatment with photosensitizing medication alongside PDT, or were treated with other PDT modalities, and/or had incomplete follow-up data were excluded. Data was collected by the clinicians responsible for the treatment in the included centers. The data on sex, age, phototype (according to Fitzpatrick), type (predominant lesion of the disease) and severity of acne according to the European and American Guidelines classification, location of lesions, duration of acne, treatment concomitant to photodynamic therapy, number of PDT sessions and their intervals, results after PDT, complications arising from PDT and follow-up were collected. All patients provided informed consent to participate in the study, and their identity was preserved in accordance with the data protection laws.

### 2.3. Treatment Protocol

All patients received the same treatment, and they were required to stop any topical treatment they were receiving one week prior to the first PDT session. The protocol started with a thorough washing of the face. Then, BF-200 ALA gel (Ameluz^®^, Biofrontera, Leverkusen, Germany) was applied in a thin layer on the affected skin, except the eyes, mouth, and nostrils. After a 30 min incubation period, the area was cleaned. The area to be treated was then illuminated using Aktilite^®^ CL 128 (Galderma, Switzerland) or the BF-RhodoLED^®^ (Biofrontera, Leverkusen, Germany). Both lamps emit a continuous, narrow-spectrum red light of 635 nm. The administered light dose was adjusted to around 4 J/cm^2^. To achieve the reduced light dose, the light intensity of the BF-RhodoLED^®^ was adjusted accordingly, and the exposure time for the Aktilite^®^ was adjusted to 3 min. The distance between the lamp and the patient’s skin was 20 cm. In case of pain, the light intensity was further reduced. Immediately after each exposure to light, a cold gel pad was applied. Please note that depending on the response and adverse events arising from each PDT session, adjustments were made to the protocol for the following sessions in a tailored treatment manner.

### 2.4. Efficacy and Safety Assessment

After each PDT session, the need for another PDT session was evaluated. Before and after each treatment, photographs were taken. Acne lesions were counted and clinically evaluated. The lesion clearance was calculated by subtracting the total number of lesions after treatment from the total number of lesions before treatment and normalizing the outcome to the total number of lesions before treatment. The lesion response categories were excellent response (clearance rate ≥ 90%), good response (clearance rate 89–60%), poor response (clearance rate 59–20%), negligible response (clearance rate ≤ 19%), and exacerbation of acne lesions (clearance rate < 0%).

Adverse effects during and after treatment, including erythema, edema, pustules, vesicles, oozing, desquamation, and hyperpigmentation, were assessed one week after each PDT session.

### 2.5. Statistical Analysis

In this study, all variables were initially analyzed descriptively using absolute and relative frequencies, mean and median, and standard deviation, where applicable. To answer more specific questions, the Chi-square test was used to assess associations between some discrete variables of interest.

## 3. Results

### 3.1. Patients

This retrospective study included 22 patients with acne vulgaris. The mean age of the population was 25.1 ± 8.9 years old. Most of the patients were female (77.3%) and had a Fitzpatrick phototype of III (63.6%). Patients had papulopustular (45.5%), nodular (27.3%), and comedonal acne (27.3%), generally of mild to moderate severity. The mean duration of acne was 5.4 ± 3.8 years. The disease manifested predominantly on the cheeks (77.3%) but was also often present on the chin and/or forehead (31.8%). Previous treatment included isotretinoin (54.5%), topical retinoids (31.8%), benzoyl peroxide (31.8%), and doxycycline (27.3%, see Table 1). Patients received PDT after the failure of these previous treatments. There was a resting period of at least 3 months prior to the initiation of the PDT treatment.

Patients received up to five PDT sessions, and most received one (36.4%) or two (27.3%) sessions (see Table 2). If more than one PDT session was performed, the interval between sessions was one month (45.5%) in most cases. About a third of the patients (36.4%; *n* = 8) received concomitant acne treatment, with topical retinoids being the most abundant (see Table 2). Follow-up was up to 36 months, but on average, 12.5 ± 10.8 months.

### 3.2. Efficacy

**Figure 1:**
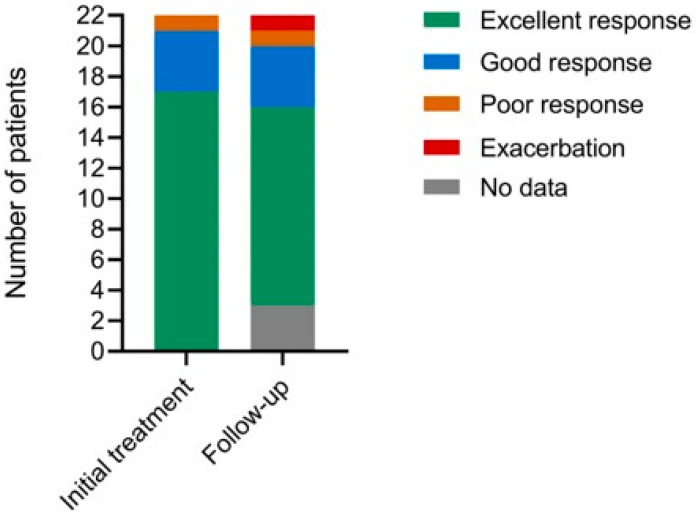
Efficacy assessment and response to treatment in patients with acne vulgaris after the PDT treatment with BF-200 ALA and at follow-up.

**Figure 2:**
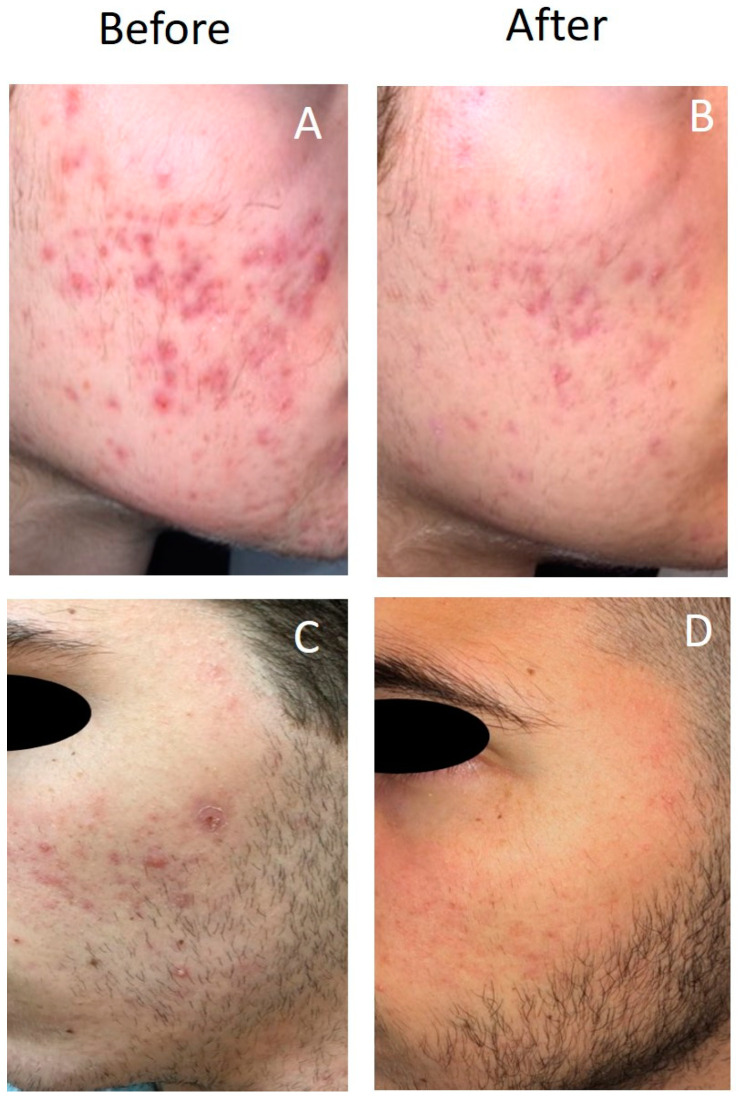
Treatment response after mild PDT with BF-200 ALA in patients with acne vulgaris. (**A**,**C**) Photographs before the treatment, left panels. (**B**,**D**) Photographs after the treatment, right panels.

Of the 22 patients in the study, 21 (95.5%) had good or excellent responses to the treatment with PDT (≥60% lesion clearance). One patient had a poor response, none exacerbated (see Table 3, Figure 1 and Figure 2). Three patients had no follow-up data. Of those patients with data (*n* = 17), 89.5% maintained disease remission at long-term follow-up. One patient had an exacerbation during follow-up.

### 3.3. Safety

Most of the patients experienced no adverse events (*n* = 16, 72.7%). Erythema was the most frequent skin reaction (*n* = 6), of which four had mild erythema, one had moderate erythema with desquamation, and one had severe erythema with associated desquamation and crusting. These adverse events were resolved within a week.

### 3.4. Associations

To assess the relationship between the use of concomitant treatment and the efficacy results, a Chi-squared test was performed. The test showed no evidence of a relationship between concomitant treatment and the efficacy results (*p* = 0.37). Furthermore, a potential association between the efficacy results and acne severity at baseline was evaluated by a Chi-squared test. Similarly, the test showed no evidence of an association (*p* = 0.19).

## 4. Discussion

With this study, we show that mild PDT using BF-200 ALA gel might be considered an effective treatment for acne. We observed a very good or good response in 95.5% of the patients treated.

Previously, a systematic review of more than 70 studies indicated that PDT has favorable results when treating acne, especially for people who do not respond well to topical or oral treatments [1]. People with more severe forms of acne may experience the most significant improvements. Amongst the different photosensitizing prodrugs used for PDT, ALA has the most extensive evidence [1]. ALA-PDT has been shown to be an effective treatment for acne, but the success rates may vary depending on the severity of the acne and the individual response to the treatment. Tao and colleagues observed a high remission rate of up to 90% using a low ALA dose (3.6%) and a high light intensity of 126 J/cm^2^ [17]. However, high remission was only achieved by a third of the patients in the study. Liu and colleagues showed a slightly lower remission rate of 78.8% with ALA-PDT [18]. The treatment protocol used here was a 1.5 h incubation of 5% ALA and illumination with 50 J/ cm^2^, indicating that good efficacy results might not be due to high light and/or drug dose.

In our study, we showed that for most patients, the very good or good results with PDT were long-lasting (over a year). Although a study performed with 20% ALA concluded that multiple treatments are better for long-lasting results [19], we are convinced that the ideal number of sessions depends on the individual skin type and the severity of the acne. This is supported by our data, as we achieved long-lasting good results with both single and multiple PDT sessions.

The National Institute for Health and Care Excellence (NICE) published a network meta-analysis in 2021 to evaluate the treatment options for acne vulgaris. PDT received a weak recommendation for people with moderate to severe acne aged 18 and over if other treatments are ineffective, not tolerated, or contraindicated. The committee acknowledged that existing evidence on light therapies is limited but promising [20]. Nevertheless, compared with standard treatments for acne vulgaris, PDT offers a rapid onset of improvement, good overall efficacy and tolerability, and comparable long-lasting results [21,22]. Efficacy might be enhanced with concomitant acne treatment, as also described briefly in the European Dermatology Forum guidelines [16]. Liu and colleagues also assessed the remission rate of PDT combined with isotretinoin and found it to be increased to 94.1%, compared with 78.8% with ALA-PDT alone [18]. In addition, ALA-PDT in combination with minocycline led to a significantly higher reduction of both inflammatory (74.4% vs. 53.3%) and non-inflammatory lesions (61.7% vs. 42.4%) 8 weeks after the end of treatment compared with minocycline treatment alone [23].

Regarding concomitant medication, the use of systemic antibiotics concomitantly with PDT in acne patients has been shown to improve patient response [23]. Specifically, in our work, four patients who were receiving minocycline (three patients) and doxycycline (one patient) had an excellent response to PDT. However, we found no association between efficacy and the use of concomitant acne treatment. Our results should be interpreted with caution, as the sample size in our study is lower than the minimum for the use of the Chi-square test. When combining treatments, possible side effects of the concomitant medication should also be considered, e.g., their photosensitizing properties [24].

In our study, most of the patients experienced no adverse events. The most common adverse event was erythema, which is consistent with the descriptions of common adverse events of PDT in general and for the treatment of acne [17,18,25,26]. This erythema is a product of the phototoxic reaction inherent to PDT. Any dermatologist who uses PDT for other indications, such as actinic keratoses or field cancerization, is usually familiar with it. Adverse reactions, as well as the efficacy of PDT, seem to be related to therapeutic parameters, including concentration of ALA, incubation time, light source, and dosage [27]. Modified and low irradiance PDT regimens generally minimize adverse reactions and usually do not limit efficacy [28,29].

We used a modified treatment protocol with a low dose of ALA, a short incubation time, and a low dose of red light. Low doses of light have also been successfully used in other studies: With a low red light dose of 15 J/cm^2^, Pollock and colleagues achieved a statistically significant reduction in inflammatory lesions with ALA-PDT compared with light treatment alone [8]. In an open study using ALA-PDT in patients with facial acne, all patients showed improvement after ALA-PDT with 13 J/cm^2^ [30].

Similarly, good results were achieved for acne by reducing the drug dose to 5% [31,32]. However, a limit to ALA dose reduction has been recently presented by He et al., showing that too low concentrations of ALA (0.1/0.5 mmol/L) might promote the growth of *C. acnes*, whereas higher doses (1.0 /2.5 mmol/L) inhibit it [33].

For the drug dose applied, it might also be considered that *C. acnes* produces porphyrins [34], and exogenous ALA can cause accumulation of either protoporphyrin or coproporphyrin and/or uroporphyrin [35]. Despite the fact that porphyrins are produced by *C. acnes*, studies have shown that exogenous ALA has to be present to achieve statistically significant improvements in acne when using PDT. Hongcharu and colleagues showed no improvement in acne vulgaris patients in ALA-alone, light-alone, or untreated sites [19]. Authors, however, used an aggressive PDT protocol, which, in their opinion, might not be necessary for sufficient inhibition of *C. acnes*. An optimized, less aggressive ALA-PDT regimen might be possible by optimizing the uptake of ALA or being more selective with the vehicle and application conditions [19]. In our study, we used the nanoemulsion BF-200 ALA, which has enhanced skin penetration characteristics compared with ALA or MAL cream [36,37,38]. In general, good results were also achieved with PDT and low doses of methyl-aminolevulinate (MAL) and/or low light doses [29,39,40]. Two studies showed that MAL-PDT significantly reduced inflammatory lesions, but the treatment had no effect on non-inflammatory lesions [41] and no significant effect on comedonal acne [9]. A small split-face study compared both ALA- and MAL-PDT for the treatment of acne [42]. The study showed no significant differences in the response rate between ALA-PDT and MAL-PDT.

Despite these promising results, the relatively small sample size of this study is a limitation that should be taken into account. Further investigations should be performed to validate these findings and elucidate the optimal protocols for the treatment of acne using PDT in clinical practice.

## 5. Conclusions

In summary, we showed in our retrospective study that PDT with BF-200 ALA gel and a low light dose is an effective and long-lasting option for the treatment of different acne types, including nodular acne. PDT was effective for all acne severity grades. We found no association between concomitant acne medication and the favorable results achieved by PDT. Most of the patients had no adverse reactions to PDT, with the most common reaction being erythema.

## Figures and Tables

**Table 1:** Patient and disease characteristics.

Patients	*N* = 22
	*n* (%)
Sex	
Male	5 (22.7%)
Female	17 (77.3%)
Age	
Mean ± SD	25.1 ± 8.9
Median (range)	23.5 (12–40)
Phototype	
I	0 (0%)
II	7 (31.8%)
III	14 (63.6%)
IV	1 (4.5%)
**Acne**	
Localization	
Neck/neckline	2 (9.1%)
Chin	7 (31.8%)
Cheeks	17 (77.3%)
Nose	6 (27.3%)
Forehead	7 (31.8%)
Acne Type	
Papulopustular	10 (45.5%)
Nodular	6 (27.3%)
Comedonal	6 (27.3%)
Acne Severity	
1	9 (40.9%)
2	6 (27.3%)
3	6 (27.3%)
4	1 (4.5%)
Duration of acne (years)	
Mean ± SD	5.4 ± 3.8
Median (range)	4.5 (1–12)
Previous treatment	
Isotretinoin	12 (54.5%)
Minocycline ^1^	1 (4.5%)
Doxycycline	6 (27.3%)
Benzoyl peroxide	7 (31.8%)
Retinoids ^1^	7 (31.8%)
Antibiotics	4 (18.2%)
Oral contraceptives	3 (13.6%)
None	1 (4.5%)

^1^ topical treatment.

**Table 2:** Study treatment characteristics.

Patients	*N* = 22
	*n* (%)
Number of PDT sessions	
1	8 (36.4%)
2	6 (27.3%)
3	5 (22.7%)
4	2 (9.1%)
5	1 (4.5%)
Interval between PDTs (months)	
1	10 (45.5%)
2	3 (13.6%)
3	1 (4.5%)
Concomitant treatment	
Yes	8 (36.4%)
No	14 (63.6%)
Type of concomitant treatment	
Retinoids	4 (18.2%)
Minocycline	3 (13.6%)
Doxycycline	1 (4.5%)
Benzoyl peroxide	1 (4.5%)
Follow-up period (months)	
Mean ± SD	12.5 ± 10.8
Median (range)	12 (0–36)

**Table 3:** Treatment response.

Patients	Initial Treatment (*N* = 22)	Follow-Up (*N* = 19)
	*n* (%)	*n* (%)
Excellent response	17 (77.3%)	13 (68.4%)
Good response	4 (18.2%)	4 (21.1%)
Poor response	1 (4.5%)	1 (5.3%)
Exacerbation	0 (0%)	1 (5.3%)

## Data Availability

The raw data supporting the conclusions of this article will be made available by the authors upon request.
